# Emergence and dynamics of self-producing information niches as a step towards pre-evolutionary organization

**DOI:** 10.1098/rsif.2017.0807

**Published:** 2018-01-17

**Authors:** Richard J. Carter, Karoline Wiesner, Stephen Mann

**Affiliations:** 1 Bristol Centre for Complexity Sciences, University of Bristol, Bristol BS8 1TS, UK; 2 Centre for Protolife Research, School of Chemistry, University of Bristol, Bristol BS8 1TS, UK; 3 School of Mathematics, University of Bristol, Bristol BS8 1TS, UK

**Keywords:** autopoiesis, protocell, information theory, prebiotic

## Abstract

As a step towards understanding pre-evolutionary organization in non-genetic systems, we develop a model to investigate the emergence and dynamics of proto-autopoietic networks in an interacting population of simple information processing entities (automata). Our simulations indicate that dynamically stable strongly connected networks of mutually producing communication channels emerge under specific environmental conditions. We refer to these distinct organizational steady states as *information niches*. In each case, we measure the information content by the Shannon entropy, and determine the fitness landscape, robustness and transition pathways for information niches subjected to intermittent environmental perturbations under non-evolutionary conditions. By determining the information required to generate each niche, we show that niche transitions are only allowed if accompanied by an equal or increased level of information production that arises internally or via environmental perturbations that serve as an exogenous source of population diversification. Overall, our simulations show how proto-autopoietic networks of basic information processors form and compete, and under what conditions they persist over time or go extinct. These findings may be relevant to understanding how inanimate systems such as chemically communicating protocells can initiate the transition to living matter prior to the onset of contemporary evolutionary and genetic mechanisms.

## 1. Introduction

Theoretical models that attempt to distinguish living from non-living systems, such as Gànti's chemoton [1,2] and Eigen & Schuster's hypercycles [3], assume the presence of replicative molecular machinery, and are constrained in their outlook as they exclude the possibility of protolife behaviour under non-replicative, non-evolutionary conditions [4]. By comparison, the theory of autopoiesis [5] postulates that a living system is distinguished by an ability to continually produce and maintain itself. As these systems properties are not necessarily contingent on the presence of a functioning genetic apparatus, the study of autopoiesis may be critical not only for understanding the transition from (geo)chemistry to protobiology on a pre-Darwinian/pre-genetic early Earth [6] but also for the laboratory-based bottom-up design and construction of synthetic cellularity [7]. Previous computational models of autopoiesis have demonstrated properties such as spatial boundary formation and self-repair in artificial chemistry systems [8–10], but a major limitation of these simulations is their reliance on the pre-existence of an ideal chemistry. While these abstract models have helped to demonstrate the concept of autopoiesis, they do not address how such chemistries come into existence, persist or compete for space, materials and energy under complex reaction conditions. In contrast, other models of autopoiesis such as algorithmic chemistry [11], algebraic chemistry [12] and matrix chemistry [13] do not specify an ideal chemistry but model the production of interacting entities to simulate the spontaneous formation of higher levels of organization.

In recent years, an alternative prebiotic evolutionary model, termed the finitary process soup [14], has been advanced. It is a model based on binary communication channels and their interaction. The channels take a single bit as input and produce a single bit as output. Since there is no stochasticity, these are deterministic input–output automata [15]. Reproduction, in this model, is represented as an interaction between two automata, with the potential of producing a new automaton. Crutchfield and Gornerup [14,16] present a detailed analysis of the structure and dynamics of this ‘soup’ of interacting automata. Interestingly, in this model, the emergence of higher level organization occurs spontaneously. Out of an initial set of 15 automata, subsets (networks) of mutually producing automata emerge. These automata networks (also called meta-machines) can be considered as self-producing, autonomous information processing entities. As such, the finitary process soup model represents a basic mechanism for the emergence of autopoiesis in an interacting population. While these findings contribute to the exploration of viable pathways to autopoiesis, they do not specifically pursue the question of how these networks form and compete, and why some networks persist over time while others go extinct.

In this paper, we extend the finitary process soup model to investigate the emergence of steady-state production networks under fixed or intermittent environmental conditions generated by changes in the degree of mixing within and influx rate into an interacting closed population of single-state automata. We find that different environmental conditions lead to different stable combinations, or networks, of mutually producing automata. We call these networks information niches, and we study their specific structural and dynamical properties. The model shows a variety of behaviour, from a small subset of mutually producing automata to a hierarchical network of automata maintaining a stable population. This is quite surprising, given that the model includes only the simplest types of input–output automata.

Furthermore, we investigated the population of automata under sudden environmental perturbations. We observed the emergence of a fitness landscape in which information niches are stable points, which the system can switch between upon perturbation. These results show how proto-autopoietic networks of basic information processors form and compete, and under what conditions they persist over time or go extinct. Thus, our model represents a mechanism for the formation of fitness landscapes under non-evolutionary conditions. These findings may be relevant to understanding how inanimate systems such as chemically communicating protocells can initiate the transition to living matter prior to the onset of contemporary evolutionary and genetic mechanisms.

## 2. Computational model and methods

### 2.1. Dynamics of information niches under fixed or intermittent environmental conditions

**Figure 1.**
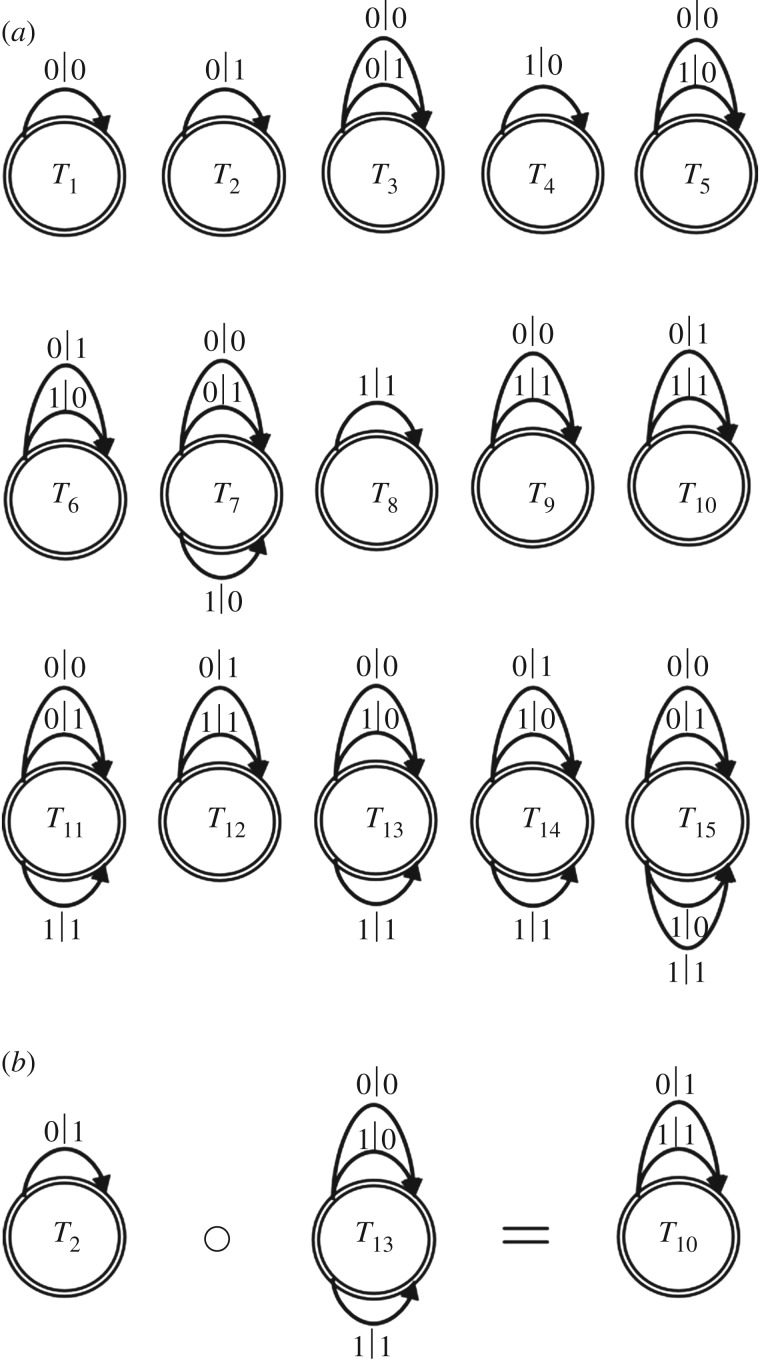
(*a*) Schematic representation showing a compositionally closed population of 15 types of single-state automata (*T*). The population includes four members that are capable of only one transition (*T*_1_, *T*_2_, *T*_4_, *T*_8_), along with six (*T*_3_, *T*_5_, *T*_6_, *T*_9_, *T*_10_, *T*_12_), four (*T*_7_, *T*_11_, *T*_13_, *T*_14_) and one (*T*_15_) that exhibit two, three and four transitions, respectively. The binary numbers on the curved arrows on the top or bottom of the circles indicate the various possible transitions; for example, *T*_3_ operates only with an input signal of 0, transducing this to either 0 (non-switched output) or 1 (switched output) with 50% probability in each case. (*b*) Scheme showing an example of the functional composition of two machines (described by the non-commutative equation, *T*_2_ ∘ *T*_13_, where ∘ is the functional composition operator) to generate *T*_10_. The three outputs from *T*_13_ are received with equal probability and transformed by *T*_2_ to produce *T*_10_, which inherits the input domain from *T*_13_ and the output range of *T*_2_. The number of possible unique binary interactions (207) is described by an interaction network (*G*) in the form of a |T| × |T| matrix; all functional compositions are members of the set of 15 types producing a compositionally closed population of interacting machines. Unsuccessful interactions between machines create the *transitionless* machine *T*_0_, which is prohibited in our model of an interacting community.

We employed a previously described computational model [14] to investigate the emergence of steady-state interacting networks and their mutual dependency within a population of interacting/replicating information processing automata. The initial population consisted of 15 types (*T*_1_ to *T*_15_) of single-state automata that act as selective communication channels capable of receiving information from a binary alphabet (*A* = {0,1}), processing the inputs using between one and four switching/non-switching binary transitions, and emitting the corresponding output in the form of a functional composition in which the sequential processing of the output from one automaton acts as the input for another (figure 1). In the original studies [14], these input–output automata were treated as a special category of finite-state transducers referred to as ɛ-machines.

Significantly, the replicating population was compositionally closed because the binary interactions between various single-state automata were unable to generate information communication channels outside the original set of 15 members.

**Figure 2.**
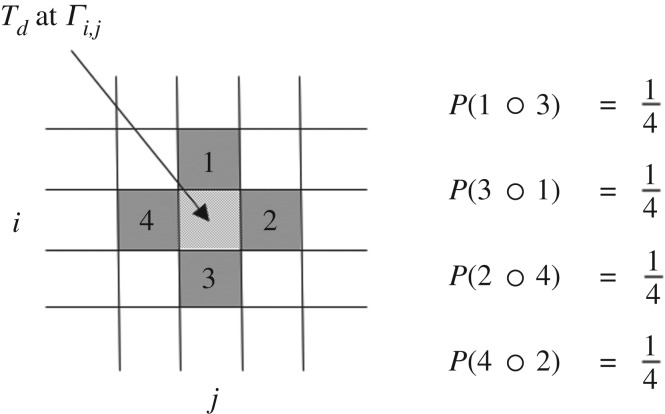
Graphic illustrating the computational model for generating internal production dynamics in a square lattice of *nxn* sites comprising single-state automata of type *T* [17]. The lattice has periodic boundary conditions, i.e. a regular toroid topology, and, as such, an automaton in the top two rows of the lattice can interact with automata directly opposite it in the bottom two rows of the lattice and vice versa. The same condition applies to an automaton on the left edge and right edge of the lattice. Spatial mixing also occurs in the same manner. An automaton (*T_d_*) on lattice site *Γ_i,j_* is chosen at random for replacement by functional composition involving types (*T_a_*,*T_b_*) selected from the sites (1,2,3,4) adjacent to *T_d_*. Only one pair of neighbours from the two possible pairwise combinations (1,3 or 2,4) is selected to interact according to the non-commutative equation *T_b_* ∘ *T_a_*, where the order of the interacting pair is selected randomly according to an equal probability of 1/4. If the functional composition generates a new automaton (*T_c_*), this replaces *T_d_* at lattice position *Γ_i,j_*, which is subsequently removed from the population. If no interaction occurs, then a transitionless machine *T*_0_ is generated such that *T_d_* is not replaced and remains on the lattice site. The new population is then subjected to different levels of spatial mixing.

An environmental context was imposed on the interacting automata by initially distributing equal numbers of the 15 types randomly across a square lattice *Γ* of *n* × *n* sites with each site occupied by an individual single-state automaton to give a population size of *N* = *n*^2^ = 90 000, which was then replicated iteratively using functional composition (figure 1*b*). The production of automata proceeded by randomly selecting a lattice site *Γ_i_*_,*j*_ whose occupying automaton (*T_d_*) may or may not be replaced by a new type *T_c_* depending on the competition between the environmental influx and internal production dynamics (figure 2). The probabilities that *T_c_* is a randomly generated automaton entering from the external environment or alternatively derived from the functional composition of two neighbouring automata were given by *Φ* and 1 − *Φ*, respectively. Production of *T_c_* by either option replaced *T_d_*, which was subsequently removed from the population to maintain a constant value for *N*. This constraint generated a survival selective pressure between different types of automata, which must be continually produced to prevent depletion from the population.

The production process was iterated for up to 1 × 10^7^ time steps to simulate the emergence of a number of distinct information niches. Changes in the structure and composition of the population were observed with increasing numbers of iterations, and this was captured at each time step by updating the frequency distribution (*f*) of the information processing types present in the emerging community. The following differential equation described the changes in *f* on each time step [17]:2.1
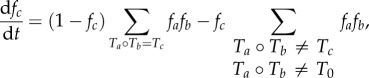
where *T_a_*,*T_b_* are the interacting machines, *T_c_* is the new automaton produced from that interaction and *f_a_*, *f_b_*, *f_c_* are their normalized frequencies of occurrence in the population. *T*_0_ is the transitionless automaton that represented an unsuccessful interaction and was prohibited in the population. Equation (2.1) determines two factors: (i) the probability of adding the automaton *T_c_* is equal to the probability of selecting two neighbours *T_a_* and *T_b_* that produce *T_c_* multiplied by the probability that the automaton that is being replaced (*T_d_*) is not the same as *T_c_* and (ii) the probability of neither *T_c_* nor *T*_0_ being produced. The invariant frequency distribution of machine types can therefore be determined by solving 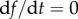. Here, discrete time is a good approximation for continuous time as only one lattice location is updated on each time step and so for large *N* (our minimum value for *N* is 90 000) this leads to a small change in the overall frequency distribution of all automata. This equation assumes that all interactions are possible on each time step, which is consistent with a well-mixed environment with no influx of automata.

Spatial mixing occurred within the population during replication by randomly selecting a lattice site and exchanging the residing automaton with another type positioned on a different lattice site along one of the cardinal directions at a distance *d* selected from a one-dimensional Gaussian distribution with variance *v* and mean = 0 and rounding *d* to the nearest corresponding lattice site. This was repeated for *c* numbers of different sites per production time step. The combination of *c* and *v* approximated diffusion within the replicating population such that when *c* → *N* and *v* → *n* the population was well mixed, while for *c* → 0 and *v* → 0 the population of automaton had very low mobility [17]. To simulate the coupling of the replicating population to changes in an external environment, randomly generated automaton types replace randomly selected automata in the population at time *t* with a probability given by *Φ*, where 0 ≤ *Φ* ≤ 1. With *Φ* = 0, no random replacement occurred and population dynamics were driven entirely by the composition of existing automata. We refer to the process of random replacement as influx to convey the notion of the movement of externally generated automata into the population. In contrast, with *Φ* = 1, the population dynamics were determined entirely by randomly generated automata entering the lattice from the external environment [14]. Twenty-five combinations of the spatial mixing (*c*, *v*) and influx dynamics (*Φ*) parameters were used to simulate a range of fixed environmental conditions to assess the impact on the production dynamics of the automaton population and the emergence of the information niches.

We investigated the effect of intermittent changes in the environmental parameters on the robustness and transition pathways of the information niches to map the fitness landscape. For this, the following modifications in environmental conditions were imposed once a steady-state niche was attained: (i) inversion of the spatial mixing parameter such that conditions contrary to those in which the niche was produced are imposed, e.g. if a niche was formed in a well-mixed environment then the environmental conditions were reset to simulate a highly restricted movement of the automata (*c*, *v =* 0); (ii) inversion of the influx setting of new machines into the lattice, e.g. if a niche was produced in the absence of any influx of automata (*Φ* = 0) then this parameter was reset to 0 < *Φ* ≤ 1; (iii) simultaneous perturbations associated with modifications (i) and (ii); (iv) introduction of a type-restricted influx (*Φ*′) of automata that were randomly selected from the specified subset of automaton types (*T*_6_,*T*_7_,*T*_9_,*T*_11_,*T*_13_,*T*_14_); and (v) simultaneous perturbations associated with modifications (i) and (iv). Perturbations on the initially produced steady-state niche were undertaken for a minimum of 1 × 10^6^ iterations, which was usually sufficient for the population to reconfigure into a new steady-state conformation. The perturbations were then removed by resetting the environmental parameters back to their original values, and changes to the population structure recorded. Consequently, the original (*primary*) niche was re-created or a new (*secondary*) niche was established by perturbing the primary niche.

### 2.2 Structure and dynamics of information niches

#### 2.2.1. Quantifying niche structure, diversity and the minimum information required for niche generation

The distribution of automaton types in a given population was structurally defined, and was responsible for the range of possible interactions. Changes in population structure, and the consequent diversity of interactions available, was quantified at each time step using the interaction network complexity (*C_μ_*(*G*)), which measured the amount of information required to describe the probability that each interaction could occur in the population contingent on the current structure of the population [14],2.2
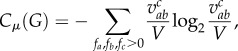
where
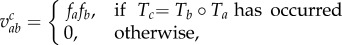



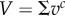
 is a normalizing factor and *f_a_*, *f_b_* are the proportion of automaton types *a* and *b* in the population, respectively. Equation (2.2) determines the likelihood of an interaction occurring to produce *T_c_* from the concentration of automata exhibiting the required functional composition. As the population evolves, some automaton types became extinct while others became more populous. As a consequence, automata produced by types that were increasing in concentration were more likely to be produced than those that were dependent on types that had become extinct. Such dynamics were reflected in the complexity of the interaction network, which reduced when some automaton types became extinct. Given that only one new automaton was produced at each time step, every interaction that occurred was *competing* with all other potential interactions. Subsequently, the probability of a specific interaction occurring was contrasted against the sum of the probability of all other possible interactions, as given by the normalization term *V*. The probability of an interaction occurring to generate an automaton was the sum of the normalized frequencies of those automata responsible for its production. This normalized probability was calculated for each machine type in the population to yield a probability distribution. The information entropy of this probability distribution then yielded the interaction network complexity *C_μ_*(*G*). As such, calculating *C_μ_*(*G*) provided a signature of the structure of the population at a given moment in time, and, when compared with the initial unstructured compositionally homogeneous population at the start of a simulation, provided a quantitative measure of the reduction in information entropy (or, conversely, the amount of order that was being created within the population) as the network system evolved into a niche.

Quantifying the minimum information required for niche generation was undertaken by defining the production threshold as a measure of the information required to describe the minimum number and type of automata that were required to be produced to create a niche. The production threshold for a niche was determined by calculating the Shannon entropy (*H*) of the frequency distribution (*X*) of each automaton type that would need to be produced within a given population,2.3
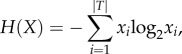
where 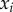 is the proportion of interactions in the population that produce automaton type *i* and *T* is the set of all automaton types. The production threshold differed between niches depending on the number of constituent automata, with lower values for those niches in which not all automaton types were present. We used this measure to compare the information required to transition between niches, elucidate how the niches transitioned in response to perturbations in environmental conditions and explain why some niches were more robust than others. In general, the production threshold and interaction network complexity were complementary. The former quantified the information required to generate a niche, whereas the latter quantified the information required to describe a niche after it had evolved to a steady-state distribution, and any interactions that remained between the automata.

#### 2.2.2. Identifying strongly connected production networks

Even in a relatively simple population of interacting entities the number of possible networks that describe all possible sequences of interactions can be significant. Identifying and examining all such sub-networks within a given population was used to identify specific networks responsible for self-organization of the population into a niche. One of the drivers of self-organizing behaviour was nonlinearity caused by positive feedback loops in the system [18], and this was manifested in an interaction network as a strongly connected cyclic topology [19,20]. We developed an algorithm to identify and categorize any sub-network structures in the interaction network (*G*) that had the motif of a *strongly connected network*, i.e. whereby a subset of automata mutually produce each other:

begin

 *W : The set of all possible combinations of sub-networks*

 *for each w in W*

  *create adjacency matrix A for w where A_i_ are outgoing edges*

  *and A_j_ are incoming edges*

   *if SUM(A_i_) ≥ 2* AND *SUM(A_j_) ≥ 2 for* EACH *node in w*

    *then add w to S*

end

This is an exhaustive algorithm that examines all possible combinations (*W*) of automaton interactions partitioned into many sub-networks (*w*) ranging in size from two to 15 automaton types. An adjacency matrix (*A*) was generated for each sub-network to describe the associated topology as a directed graph [21] with nodes and directed edges indicative of an individual automaton type and which automata interact to produce other automaton types, respectively. The adjacency matrix of each sub-network was tested for the characteristic of mutual production between members (i.e. a cycle), whereby each automaton (node) in the sub-network must have: (i) a minimum in-degree of two edges, implying that it is produced by at least one interaction of automata in the sub-network apart from with itself (self-replication), and (ii) a minimum out-degree of two edges, implying that the automaton produces one other automaton apart from itself within the sub-network. A sub-network was considered to be a candidate for a strongly connected network only if all constituent automata fulfil these criteria. The set of candidate strongly connected networks (*S*) was then examined for dynamic stability.

#### 2.2.3. Determining dynamically stable networks

To identify dynamically stable networks we numerically solved 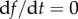 of equation (2.1) for each strongly connected network (*S*) identified in the interaction network (*G*). All sub-networks in *S* were examined for dynamic stability and the automaton types in the numerical simulation were restricted to those present in the sub-network under consideration. Networks whose production dynamics resulted in extinction of any of the constituent members, or which created new information processors that were not original members of the network, were deemed unstable.

## 3. Results

### 3.1. Emergence and properties of primary information niches under fixed environmental conditions

**Figure 3.**
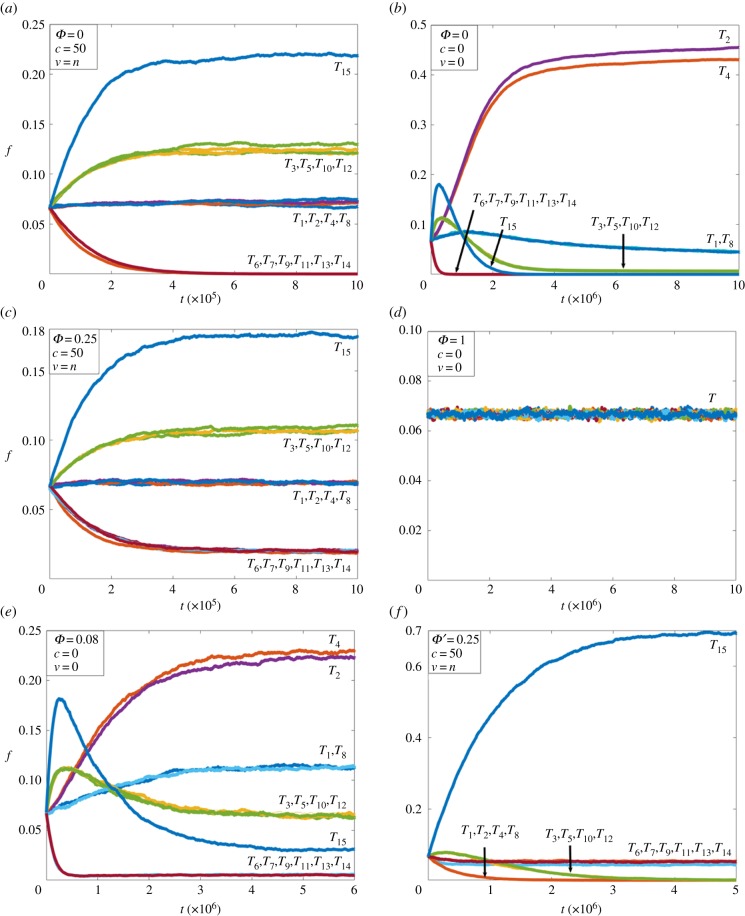
Plots of frequency distributions (*f*) against iteration time step (*t*) for interacting populations of automata under different simulated fixed environmental conditions. The simulations show the evolution of six distinct information niches comprising steady-state networks of selected and clustered information processing channels from an interacting population consisting of 90 000 single-state automata distributed at *t* = 0 equally across 15 different types (shown in different colours) and subjected to three different environment inputs. (*a*) Niche *A*: population production dynamics in an environment with high spatial mixing of automata (1 ≤ *c* ≤ *N*, 1 ≤ *v* ≤ *n*) and with no influx of randomly generated automata (*Φ* = 0) showing extinction of six automaton types and the emergence of a steady-state distribution of nine survival types arranged into three distinct clusters with one, four or four members after *t* = 4 × 10^5^ time steps. (*b*) Niche *B*: extinction and steady-state survival of 11 and four binary automaton types, respectively, under a highly immobile environment exhibiting no diffusivity (*c* = 0, *v* = 0) and no randomly generated influx (*Φ* = 0). The survivors are arranged in three sub-groups containing one, one or two members. (*c*) Niche *C*: population production dynamics across a range of mixing conditions (0 ≤ *c* ≤ *N*, 0 ≤ *v* ≤ *n*) and subjected to a considerable rate of influx of randomly generated automata (0.25 ≤ *Φ* ≤ 0.9). All automaton types survive to produce a heterogeneous population structure comprising four steady-state clusters consisting of one, four, four and six members. The population is structured similarly to niche *A* except that six members no longer become extinct. (*d*) Niche *D*: under all mixing conditions (0 ≤ *c* ≤ *N*, 0 ≤ *v* ≤ *n*) and with *Φ* *>* 0*.*9 the population dynamics are dominated by the influx of new randomly generated automata from the environment such that the population remains unstructured and compositionally homogeneous over 1 × 10^5^ iterations. (*e*) Niche *E*: with no diffusive mixing on the lattice (*c* = 0, *v* = 0) and with a very low influx rate (0 < *Φ* < 0.1); the population is structurally similar to niche *B* except that three sub-groups of automaton types ((*T*_15_), (*T*_3_,*T*_5_,*T*_10_,*T*_12_) and (*T*_6_,*T*_7_,*T*_9_,*T*_11_,*T*_13_,*T*_14_)) no longer go extinct. (*f*) *Niche F:* with restricted automaton influx (*Φ*′ = 0.25) and under a range of mixing conditions (0 ≤ *c* ≤ *N*, 0 ≤ *v* ≤ *n*) the population evolves to three groups of automata (*T*_15_), (*T*_6_,*T*_7_,*T*_9_,*T*_11_,*T*_13_,*T*_14_) and (*T*_3_,*T*_5_,*T*_10_,*T*_12_), with the group (*T*_1_,*T*_2_,*T*_4_,*T*_8_) going extinct.

Twenty-five environments were simulated by setting unique combinations of the spatial mixing (*c*, *v*) and influx dynamics (*Φ*) parameters in the range of 0 ≤ *c* ≤ *N*, 0 ≤ *v* ≤ *n* and 0 ≤ *Φ* ≤ 1 for an evolving population of 90 000 interacting automata distributed equally across 15 different types. The emergence of steady-state network configurations (information niches) under fixed environmental conditions typically required between 1 × 10^6^ and 1 × 10^7^ iterations. After every iteration, the changes in frequency (*f*) of each automaton type were determined until steady-state conditions were attained. Significantly, six distinct primary information niches *(A–F)* comprising strongly connected components of self-producing communication channel networks were obtained (figure 3).

#### 3.1.1. Influence of spatial mixing and non-diffusivity

Niche *A* consisted of a steady-state network of nine automata that emerged from a well-mixed population (1 ≤ *c* ≤ *N*, 1 ≤ *v* ≤ *n*) in the absence of an influx of randomly generated automata (*Φ* = 0). Six automata became extinct (*T*_6_, *T*_7_, *T*_9_, *T*_11_, *T*_13_, *T*_14_) and the remaining nine types differentiated into three distinct clusters exhibiting no growth (*T*_1_, *T*_2_, *T*_4_, *T*_8_), slow growth (*T*_3_, *T*_5_, *T*_10_, *T*_12_) and fast growth (*T*_15_), all of which reached steady-state frequencies after *t* = 4 × 10^5^ time steps (figure 3*a*). *T*_15_ was produced from 35 interactions and was therefore the most frequently produced automaton. In comparison, automaton types in the slow and no growth clusters were generated from 21 or 15 interactions, respectively, while those that became extinct were produced from only eight interactions involving the (*T*_7_, *T*_11_, *T*_13_, *T*_14_) (six interactions) and (*T*_6_, *T*_9_) (two interactions) sub-groups. Interestingly, extinction of the six automata resulted in a drastic reduction in the number of interactions in the population from 207 to 63 interactions, which were then responsible for producing each of the remaining automata at an equal rate (seven interactions per automaton) and establishing steady-state conditions within the population.

In contrast, simulations of the population production dynamics under fixed conditions of no spatial mixing (*c* = 0, *v* = 0) and no randomly generated influx (*Φ* = 0) produced niche *B*, which comprised a four-automaton steady-state network consisting of types *T*_1_, *T*_2_, *T*_4_, and *T*_8_ (figure 3*b*). The population dynamics initially mirrored those observed for a well-mixed environment (niche *A*), but then exhibited a major transition at *t* = 2 × 10^5^ after which the initial growth of *T*_15_ and the (*T*_3_, *T*_5_, *T*_10_, *T*_12_) group was replaced by a rapid decrease in their frequency such that these automata became extinct after approximately 3 × 10^6^ iterations. As a consequence, the (*T*_1_, *T*_2_, *T*_4_, *T*_8_) group, which exhibited no growth in a well-mixed environment (niche *A*), differentiated into fast growing and non-growing populations of *T*_2_ and *T*_4_, and *T*_1_ and *T*_8_, respectively, with the (*T*_2_, *T*_4_) pair occupying approximately 85% of the final population of niche *B* produced in the absence of spatial mixing. Under these conditions, interactions between the automata were spatially restricted such that short-range interactions dominated the population dynamics. As a consequence, two mechanisms were responsible for the fast growth of *T*_2_ and *T*_4_ in niche *B*: (i) independent interactions between *T*_2_ or *T*_4_ with a range of other automata gave rise to self-replication, or alternatively to production of *T*_1_ and *T*_8_, which subsequently interacted with various other automata to generate *T*_2_ and *T*_4_ and (ii) local concentrations of *T*_2_ and *T*_4_ produced a spatial cluster (defined as a contiguous square area of the lattice consisting of nine *T*_2_ or *T*_4_ automata), which acted as a nucleation domain for protected outgrowth.

#### 3.1.2. Influence of influx dynamics

Having simulated the influence of spatial mixing and non-diffusivity on niche formation, we next investigated the effect of introducing an influx of randomly generated automata into an interacting population of automata under a range of mixing conditions. In the presence of both spatial mixing and significant influx dynamics (0 ≤ *c* ≤ *N*, 0 ≤ *v* ≤ *n* and 0.25 ≤ *Φ* ≤ 0.9), the emerging steady-state population (niche *C*) was structured similarly to niche *A* except that the (*T*_6_, *T*_7_, *T*_9_, *T*_11_, *T*_13_, *T*_14_) group no longer became extinct (figure 3*c*). As a consequence, all 15 automaton types survived to produce a heterogeneous population structure comprising four steady-state clusters consisting of (*T*_6_, *T*_7_, *T*_9_, *T*_11_, *T*_13_, *T*_14_) with a decreased frequency, (*T*_1_, *T*_2_, *T*_4_, *T*_8_) with constant frequency, and (*T*_3_, *T*_5_, *T*_10_, *T*_12_) and *T*_15_, which exhibited slow and fast growth, respectively. In contrast, simulations of the population production dynamics under spatial mixing (0 ≤ *c* ≤ *N*, 0 ≤ *v* ≤ *n*) and with a very high influx of randomly generated automata (0.9 < *Φ* ≤ 1; niche *D*) indicated that under these conditions the population dynamics were dominated by the influx rate from the environment. As a consequence, the population had no memory of previous interactions, and therefore remained unstructured with a composition uniformly distributed over all 15 automaton types even over 1 × 10^5^ iterations (figure 3*d*). We simulated the population production dynamics under conditions of no diffusive mixing on the lattice (*c* = 0, *v* = 0) and with a very low influx rate (0 < *Φ* < 0.1). The resulting niche *E* was structurally similar to niche *B* (*c* = 0, *v* = 0; *Φ* = 0) but showed no automaton extinctions.

Finally, we simulated the population dynamics under a type-restricted influx (*Φ*′ = 0.25) comprising randomly generated automata drawn from a specified subset of automaton types (*T*_6_, *T*_7_, *T*_9_, *T*_11_, *T*_13_, *T*_14_). This specific subset was chosen as it represented the automaton types that were most frequently depleted from the population, or in the case of niches *A* and *B* became extinct. Thus, by restricting the environmental influx to this subset, we not only increased their concentration in the environment but also increased the probability that an automaton type from this subset would be re-introduced into the population during the simulation. As a consequence, rapid decay and extinction of (*T*_1_, *T*_2_, *T*_4_, *T*_8_), slow decay and extinction of (*T*_3_, *T*_5_, *T*_10_, *T*_12_) and rapid growth of *T*_15_ were observed to produce niche *F*. Niche *F* was structured into two groups consisting of a dominant automaton (*T*_15_) that occupied 70% of the population, along with a constant concentration of the (*T*_6_, *T*_7_, *T*_9_, *T*_11_, *T*_13_, *T*_14_) cluster, which was sustained by the limited influx dynamics into the lattice. Significantly, *T*_15_ exhibited rapid growth because it was the only automaton produced (via eight interactions) by the restricted subset of influx automata (*T*_6_, *T*_7_, *T*_9_, *T*_11_, *T*_13_, *T*_14_), and was the only self-replicator in the population.

#### 3.1.3. Niche landscape and niche construction

**Figure 4.**
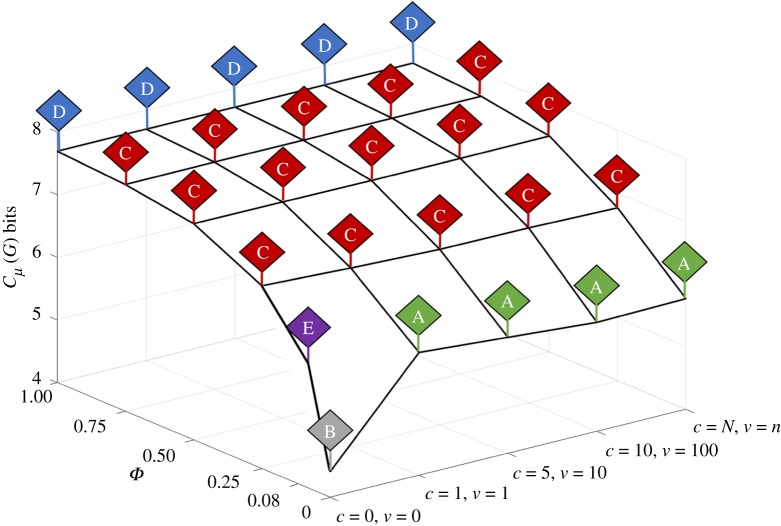
Three-dimensional map of the interaction network complexity *C_μ_*(*G*) against environmental parameters (*c*, *v*, *Φ*) showing the information niche landscape. The information niches reside at different levels of *C_μ_*(*G*). Note the prevalence of niche *C*. Niche *F*, which is produced under compositionally restricted influx (*Φ*′ = 0.25), is not shown. There is little sensitivity to changes in the interaction network complexity measured for a wide range of values for *c* and *v*. In general, spatial mixing has a mild effect on population structure, and given that the interaction network complexity is contingent on structure, results in minor changes to the interaction network complexity.

Niche *C* was predominant across a wide range of fixed conditions of spatial mixing and random influx, indicating that the network constellation producing the distinctive four clusters was extremely robust. In contrast, niches *A* and *D* were produced under a limited set of conditions (1 ≤ *c* ≤ *N*, 1 ≤ *v* ≤ *n*; *Φ* = 0 and 0 ≤ *c* ≤ *N*, 0 ≤ *v* ≤ *n; Φ* *>* 0*.*9, respectively), and *B* represented a singularity at *c* = 0, *v* = 0; *Φ* = 0. The corresponding information landscape was mapped by plotting the interaction network complexity values (*C_μ_*(*G*)) for niches produced under different environmental conditions (figure 4).

A niche with a higher interaction network complexity has more interactions and a more uniform distribution of automata and hence there is more uncertainty over what the next automaton to be produced will be. By contrast, there is less uncertainty in a lower complexity niche for the opposite reasons, i.e. fewer possible interactions and a non-uniform population and therefore more certainty over which automata are likely to be produced. An alternative interpretation is that more complex niches host a greater degree of competition between automata to reproduce due to each automaton having a lower probability of being produced than an automaton in a less complex niche (as measured by a lower interaction network complexity).

The initially unstructured and uniformly distributed population at *t* = 0 had a *C_μ_*(*G*) value of 7*.*7 bits that represented all 207 possible interactions, and this reduced to 5*.*8 bits for niche *A* (63 interactions) and to 2*.*6 bits for niche *B* (eight interactions), indicative of higher levels of structuration particularly for niche *B.* In contrast, the *C_μ_*(*G*) value for niche *C* was 7.0 bits, which represented all 207 interactions and a small decrease in complexity (−0*.*7 bits) due to structuring of the population into four clusters. As niche *D* contained no changes in the frequency distribution of the original population, the *C_μ_*(*G*) value remained at 7*.*7 bits. Niche *E*, which had a similar spatial lattice structure to niche *B* but with inclusion of all automaton types in the population, had a *C_μ_*(*G*) value of 6 bits. This represented all 207 possible interactions but with more and less structure than niche *C* and niche *B*, respectively. In general, the rates of formation of the information niches were decreased as the rate of spatial mixing decreased and/or the influx rate of new randomly generated automata increased. For example, growth of the (*T*_2,_ *T*_4_) group in niche *B* was reduced as 0 *< Φ* ≤ 0*.*1 and disappeared with 0.1 < *Φ* ≤ 1. This indicated that increasing the number of automaton types persisting in the population due to a continuous influx from the environment (*Φ* *>* 0) destabilized the onset of structuration and the concomitant emergence of steady-state networks. In contrast, at *Φ* = 0, 11 automaton types became extinct in niche *B*, which reduces the robustness of the network with respect to its ability to self-generate.

**Figure 5.**
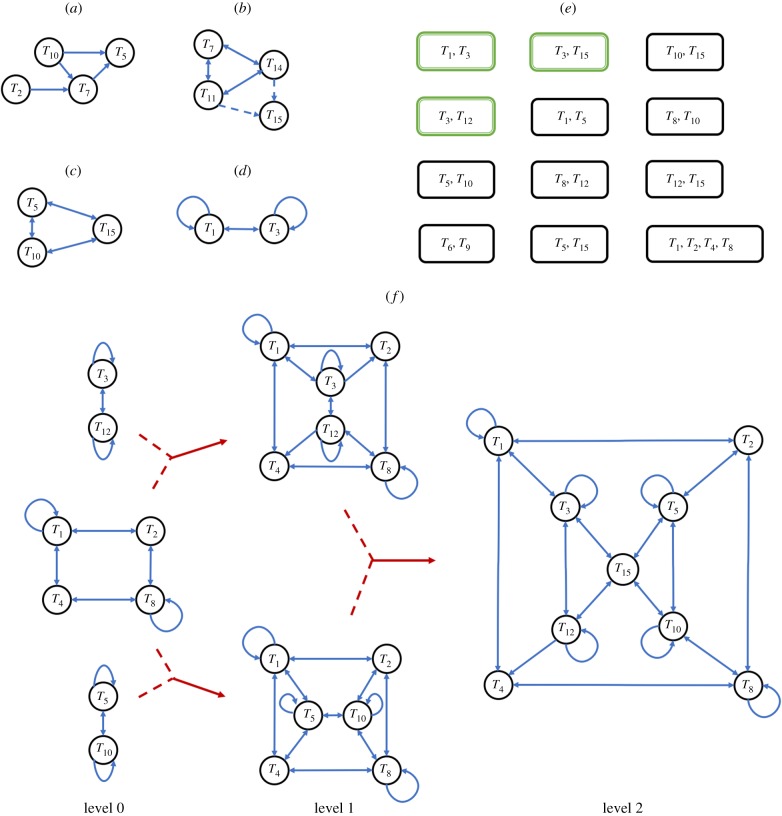
(*a–d*) Examples of the different types of network topologies generated from all combinations of interactions between automata in order of increasing specialism. (*a*) The most general topology (7702 identified types). The topology consists of a directed network in which each node represents an automaton and edges (arrows) signify that the automaton is involved in the production of the automaton positioned at the termination of the edge. For example, automata *T*_2_ and *T*_10_ interact to produce *T*_7_, which interacts with *T*_10_ to produce *T*_5_. In this example, the algorithm identifies the three networks {*T*_2_, *T*_7_, *T*_10_}, {*T*_5_, *T*_7_, *T*_10_} and {*T*_2_, *T*_5_, *T*_7_, *T*_10_}. (*b*) Network with *apparent* strongly connected components (100 identified types), indicating that the constituent automata mutually produce each other; however, over time automata are also produced outside of the network (dashed arrows leading from *T*_11_ and *T*_14_), or the system becomes dynamically unstable because of competition within the same network that leads to extinction of one or more of its members. Edges with double arrows indicate that the connected automata are involved in producing each other. (*c*) Example of a strongly connected network (17 identified types) that only produces automata within the network, is dynamically stable and can be reduced into smaller sub-networks. (*d*) Example of a strongly connected network (12 identified types) that is dynamically stable and irreducible (elementary networks). A curved arrow indicates that the automaton is involved in its own production. (*e*) All 12 elementary networks of a one-state automaton population. Some automata are produced by more than one network and this is highlighted for *T*_3_ (green boxes). Multiple pathways to producing the same automaton (redundancy) confers a degree of robustness to the continued production of an automation even if an elementary network decays due to the extinction of one of its constituents. By comparison, *T*_6_ and *T*_9_ are only produced by one elementary network and these automata often become extinct. Niche *A* consists of all elementary networks except {*T*_6_, *T*_9_}; niche *B* consists of only one network {*T*_1_, *T*_2_, *T*_4_, *T*_8_}; and niche *C* consists of all of the elementary networks. (*f*) An example of a pathway to the bottom-up hierarchical construction of niche *A* based on the integration of three elementary networks (level 0) that combine to form two larger networks (level 1), which become embedded at level 2. Note that *T*_15_ is only produced when the level 1 networks are combined. Double arrows indicate that the associated automata are involved in producing each other and curved arrows indicate an automaton that is involved in self-production.

We executed an algorithm to identify interaction networks in a one-state automaton population that had strongly connected topologies characteristic of mutual production. The algorithm generated 7831 interaction networks ranging in size from two to 15 automata and exhibiting different levels of specialism (figure 5*a–d*). A subset of 129 networks was identified as strongly connected, implying that they were closed under composition. Of these, 29 were dynamically stable, i.e. under dynamical conditions each automaton in the network continued to be produced at a rate that no single automaton was over-produced (leading to complete dominance) or under-produced (leading to decay and ultimately extinction) within the population.

Such networks are similar to meta-machines [16], and endured indefinitely unless subjected to changes in the environmental conditions that disrupted the population dynamics. Of the 29 closed and stable networks, niche *A* (high spatial mixing (1 ≤ *c* ≤ *N*, 1 ≤ *v* ≤ *n*) and no intake dynamics (*Φ* = 0)) contained 28 (the closed and stable network {*T*_6_, *T*_9_} became extinct with time), while niche *B* with no lattice diffusion and no random influx (*c* = 0, *v* = 0; *Φ* = 0) contained only one. In contrast, niche *C*, which emerged under a wide range of population mixing and influx conditions (0 ≤ *c* ≤ *N*, 0 ≤ *v* ≤ *n*; 0.25 ≤ *Φ* ≤ 0.9), contained all 29 dynamically stable strongly connected networks. As expected, niche *D* produced under high levels of spatial mixing (0 ≤ *c* ≤ *N*, 0 ≤ *v* ≤ *n*) and very high influx (*Φ* *>* 0*.*9) did not contain any stable networks.

We also searched for stable networks of production that were not only closed under composition but also irreducible. Production networks were irreducible if removal of one automaton resulted in dynamic instability that led to the decay of the network to a single automaton. Of the 29 dynamically stable networks, a subset of 12 networks termed *elementary networks* was identified as being closed, stable and irreducible (figure 5*e*). Niches *A*, *B*, *C* and *D* contained 11, 1, 12 and 0 elementary networks and niches *E* and *F* contained 12 and 0 elementary networks, respectively. Significantly, there was an association between niches with a higher number of elementary networks and their persistence across a range of environmental conditions, e.g. niches *A* and *C* collectively occupied approximately 75% of the information landscape (figure 4). While this was not a universal finding—niche *E* occurred only once and this was due to its formation exclusively in a low influx and low diffusive environment—it did suggest a degree of robustness conferred on a niche courtesy of the presence of more than one elementary network.

The presence of the elementary network {*T*_6_,*T*_9_} in niche *C* was a direct consequence of the influx of randomly generated automata from the environment as this elementary network did not persist in the absence of any influx (niche *A*). Significantly, information niche *A* was constructed from a hierarchical organization in which the successive combination of elementary networks (level 0) produced intermediate networks (level 1), which in turn were integrated and embedded in a higher-order structure (level 2) (figure 5*f*). There were 15 construction pathways by which the higher-order network produced niche *A*, with each pathway the result of a unique combination of elementary and intermediate networks. Each network in the hierarchical organization was closed and dynamically stable. In contrast, niche *B* with a single elementary network was non-hierarchical.

The pathway to niche *C* involved the same elementary networks as niche *A*, except that in the presence of an influx of randomly generated automata (0 < *Φ* ≤ 0.9) the group (*T*_6_, *T*_7_, *T*_9_, *T*_11_, *T*_13_, *T*_14_) became organized into strongly connected networks that were dynamically unstable, and were therefore not part of the network hierarchy. Significantly, niches *A* and *C* consisted of automata that were each produced by at least two elementary networks, indicating a level of redundancy in the organization (figure 5*f*); for example, deconstruction of niche *A* indicated that the redundancy at level 1 involved decomposition of the intermediate networks into a subset of six ({*T*_1_, *T*_3_}, {*T*_1_, *T*_5_}, {*T*_3_, *T*_12_}, {*T*_5_, *T*_10_}, {*T*_8_, *T*_10_}, {*T*_8_, *T*_12_}) of the total of 12 elementary networks.

### 3.2. Dynamics of information niches under environmental perturbations

**Figure 6.**
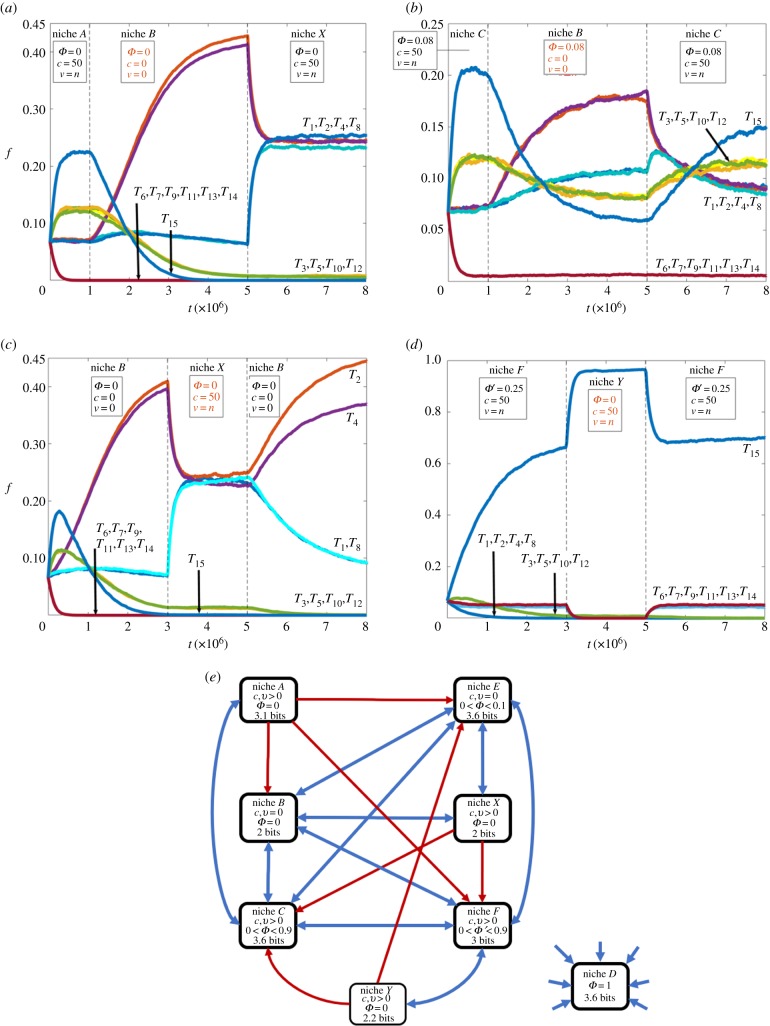
Plots of frequency distributions (*f*) against iteration time step (*t*) showing the population dynamics when subjected to intermittent and extreme changes in environmental conditions (red text). (*a*) Primary information niche *A* was established after 1 × 10^6^ iterations, and then the lattice diffusivity reduced to zero. Niche *B* emerged within 5 × 10^6^ iterations and subsequently resetting the parameters to their original values formed a new secondary niche *X*, which was distinguished by two groups of automata (*T*_1_,*T*_2_, *T*_4_, *T*_8_) and (*T*_3_, *T*_5_, *T*_10_, *T*_12_). (*b*) Similarly, primary niche *C* was established after 1 × 10^6^ iterations and then the lattice diffusivity reduced to zero leading to the reversible emergence of niche *E* after 5 × 10^6^ iterations; resetting the parameters to their original values re-created niche *C*. (*c*) Formation of the secondary niche *X*; niche *B* formed after 3 × 10^6^ iterations and then the lattice diffusivity was increased for 2 × 10^6^ iterations to generate niche *X* after which the perturbation was removed and the population transitioned back to niche *B*. (*d*) Primary niche *F* was established under type-restricted automaton influx (*Φ*′ = 0.25), and then the population perturbed by removing the intake restriction to produce the homogenized secondary niche *Y* comprising the self-replicator *T*_15_; resetting the parameters led to the reverse transition back to niche *F*. (*e*) Information niche transition diagram; each box represents a niche and the associated environmental parameters indicate the conditions under which the information niche forms, and the production threshold of the niche in binary digits. Arrows between niches indicate possible transitions and whether they are irreversible (red single arrows) or reversible (blue double arrows). Niche *D* is a special case as it represents an unstructured, uniformly distributed population, which can be produced from perturbing all primary and secondary niches by setting *Φ* = 1.

To investigate the influence of environmental perturbations on the robustness and possible transitions of the primary information niches we simulated the response in the population dynamics to intermittent changes in the parameters *c*, *v*, *Φ* and *Φ*′ (figure 6). These parameters were varied to simulate five types of perturbation that were imposed on each primary niche (niches *A–F*): (i) switching of lattice diffusivity to a value opposite to that initially associated with niche formation; (ii) switching of the influx rate to one of four possible values (*Φ* = 0, 0 < *Φ* < 0.1, 0.1 ≤ *Φ* ≤ 0.9 and 0.9 < *Φ* ≤ 1), which in each case corresponded to a parameter opposite to that initially associated with niche formation; (iii) application of (i) and (ii) concurrently; (iv) restricting the influx composition from a random selection of 15 automaton types to a confined group of six specified automata (*T*_6_, *T*_7_, *T*_9_, *T*_11_, *T*_13_, *T*_14_) at a rate *Φ*′ = 0.25; and (v) application of (i) and (iv) concurrently. In general, the results indicated that imposing environmental perturbations on the primary niches produces transformations in the internal structure of the population through the growth or decay in various automaton types, which under certain conditions (introducing diffusive mixing into niche *B*/*E* or removing influx into niche *F*) generated two new secondary niches (niche *X* and niche *Y*). We then perturbed these secondary niches by re-setting the environmental parameters to those initially used for the formation of the associated primary niche to assess the reversibility of the niche transitions across the information landscape (figure 6*c–d*).

In total, 39 transitions between six primary niches and two secondary niches were identified (figure 6*e*). In some cases, the transitions were unidirectional. For example, niches *A* and *C* were established after 7.5 × 10^5^ iterations under well-mixed conditions with no or low intake dynamics (*c* = *N*, *v* = *n*; *Φ* = 0 or *c* = *N*, *v* = *n*; *Φ* = 0.08), respectively, and then subjected to an extreme perturbation by switching the lattice diffusivity to zero (*c* = 0, *v* = 0). As a consequence, over 1.25 × 10^6^ iterations niches *B* and *E* emerged in each population, respectively, with *T*_15_ experiencing a rapid decay while *T*_2_, *T*_4_ underwent fast growth. Once niches *B* or *E* were fully established, we re-adjusted the parameters to their original values, and assessed how the emerging populations responded. Niche *C* was re-established within 1 × 10^6^ iterations, indicating that the *C* to *E* transition was reversible across the information landscape under the imposed environmental conditions, while niche *A* was not re-established from niche *B*. Instead, niche *B* transitioned into a new niche (niche *X*), which consisted of eight automaton types clustered into two groups (*T*_1_, *T*_2_, *T*_4_, *T*_8_) and (*T*_3_, *T*_5_, *T*_10_, *T*_12_) (figure 6*a*,*b*). Secondary niche *X* was also produced from niche *E* by introducing lattice diffusivity into the simulations. Increasing the number of long-range interactions within the highly structured populations of niches *B* and *E* eliminated the *T*_2_ and *T*_4_ domains such that the production dynamics were dominated by the elementary network {*T*_1_, *T*_2_, *T*_4_, *T*_8_}, which produced each of its members with equal probability. This led to a transient period with a reduction in the number of *T*_2_ and *T*_4_ automata and corresponding increase in the number of *T*_1_ and *T*_8_ automata until a new steady state was reached after approximately 5 × 10^5^ iterations (figure 6*c*).

Secondary niche *Y* was generated by perturbation of primary niche *F*, which was produced under restricted influx conditions (*Φ*′ = 0.25) via switching off the partial influx of new automata (*Φ*′ = 0) (figure 6*d*). Under the new environmental conditions, the *T*_15_ frequency, which comprised approximately 70% of the population of niche *F*, increased rapidly to almost 100% in niche *Y* to produce a homogenized population. This was principally because (i) *T*_15_ could be generated from 21 interactions including a high level of self-replication and (ii) the clusters (*T*_7_, *T*_11_, *T*_13_, *T*_14_) and (*T*_6_, *T*_9_) were each produced from only six and two interactions, respectively, and collectively did not form a closed and stable network of production.

The production threshold was calculated for each niche and the loss or gain of information between niches undergoing reversible or non-reversible transitions examined. As shown in figure 6*e*, the production threshold of primary niches *C*, *D* and *E* was 3.6 bits, niche *A* was 3.1 bits, and niches *F* and *B* was 3 bits and 2 bits, respectively. The production thresholds for the secondary niches *X* and *Y* were 2 and 2.2 bits, respectively. As the production threshold relates to how much information is required for a niche to persist via the continual production of certain automata, in general transitions within the niche landscape occurred when there was a reduction or no significant change in the information content. However, transitions that resulted in a loss of information and a subsequent reduction in the production threshold of the population were irreversible unless sufficient information was added from the environment. For example, niche *A* (3.1 bits; 0 < *c* ≤ *N*, *0 < v* ≤ *n*; *Φ* = 0) transitioned to niche *B* by setting the lattice diffusivity to zero (*c*, *v* = 0), which resulted in a reduced information content (2 bits) because extinction of *T*_15_ reduced the number of possible interactions in niche *B*. Re-setting the parameters to enable lattice diffusivity (0 < *c* ≤ *N*, *0 < v* ≤ *n*; *Φ* = 0) did not re-establish niche *A* because increasing spatial mixing did not provide additional information content (*T*_15_ was irredeemably lost from the population). Instead, niche *B* transitioned into niche *X* (2 bits) that had the same information content as niche *B* but a different steady-state configuration. Indeed, the only way to re-gain lost information was through an influx of automata from outside the population by increasing the *Φ* parameter. Thus, the transition from niche *C* (0 < *c* ≤ *N*, *0 < v* ≤ *n*; 0 < *Φ* < 0.9) to *B* (*c*, *v* = 0; *Φ* = 0) was reversible because the initial perturbation step was linked with a reduction of information from 3.6 to 2 bits (figure 6*e*), and the return pathway associated with an increase in new information due to the re-established environmental influx of automata.

Based on the above analysis, the robustness of each niche within the information landscape was observed to be dependent on the environmental conditions under which it was formed, and the nature of any subsequent perturbations. In particular, niches that were generated under zero influx conditions resulted in the extinction of six (*T*_6_, *T*_7_, *T*_9_, *T*_11_, *T*_13_, *T*_14_) of the 15 types of automata, while all the automaton types were retained in niches constructed under high influx conditions. Robust niches such as niches *C*, *D*, *E* and *F* could recover from any type of perturbation, and were associated with high information environments characterized by the presence of lattice diffusivity (0 < *c* ≤ *N*, *0 < v* ≤ *n*) and some environmental influx (0 < *Φ* < 0.9). Conversely, niches that were less robust were produced in low information environments characterized by zero diffusivity (*c*, *v* = 0) and no influx of automata (*Φ* = 0).

## 4. Conclusion

**Figure 7.**
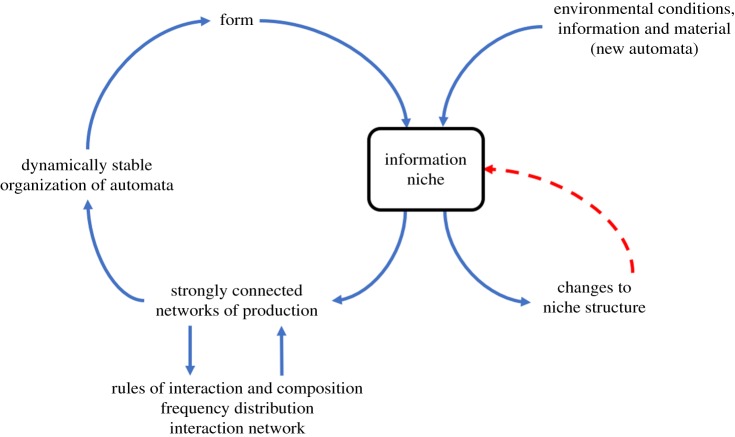
The information niche as a nexus: interactions between automata according to intrinsic (rules of composition and competition) and extrinsic (changes in frequency distribution and interaction network) factors generate strongly connected networks of production that evolve to dynamically stable organizations depending on the coupling with environmental factors (spatial mixing/intake dynamics) to form an information niche. The niche serves as a nexus as it is responsive to external factors such as changes in environmental conditions, information content and input dynamics, as well as to internal changes in structure. The circularity generates a compositionally closed system with the niche acting in a top-down manner to influence the networks of production, thereby maintaining its own identity and demonstrating proto-autopoietic properties.

We have examined a self-producing system in a pre-evolutionary/pre-genetic scenario by extending the finitary process soup model [14] to investigate the influence of environmental conditions and perturbations on the dynamics and emerging organizational complexity of an interacting population of single-state information processing entities (automata). Our simulations indicate that dynamically stable strongly connected networks of mutually producing automata emerge under specific environmental conditions associated with changes in the degree of spatial lattice mixing and influx dynamics. The emergence of a limited number of these information niches suggests an underlying fitness landscape, which sculpts the self-organizing community of interacting automata into a self-referential system that is contingent on the interplay of internal and external population production dynamics (figure 7). In this perspective, the information niche represents a nexus between four key processes: (i) the mutual production of automata and formation of closed and stable networks, (ii) emergence of a hierarchical interaction network structure, (iii) onset of dynamic stability in the networks of production, and (iv) redundancy within the population and interaction network.

Our simulations indicate that an information niche was more robust and viable with increasing levels of redundancy, as each automaton was produced by at least two different and independent elementary networks, and the associated modularity enables effective niche recovery when subjected to extreme environmental perturbations. Among the fitness landscape, niches *C* and *E* are able to reconstruct when subjected to fluctuating environmental parameters principally due to modulating the interface with the environment by coupling of the internal production dynamics to the randomly generated intake of new automata. Significantly, reversible niche transitions are only allowed if accompanied by an equal or increased level of information production. In some cases, the environmental perturbation generates additional information that drives the niche transition, and as such acts as an exogenous source of diversification of the population. Thus, taken together our simulations show that characteristics indicative of mutual production and redundancy confer resilience on the dynamics and emerging organizational complexity of interacting/replicating populations of simple information processing entities.

Within a more general context, our model describes a basic mechanism for coupling environmental parameters into a community of interacting objects that function as communication channels, and therefore offers a new approach for studying the onset of autopoiesis within both a prebiological scenario and bottom-up synthetic biology context. We demonstrate that the emergence of information niches occurs without the introduction of novel forms into an environmental fitness landscape, suggesting that communities of interacting entities such as chemically active synthetic protocells [22,23] could become hierarchically structured and dynamically stable over time even in the absence of evolution. Such observations provide insights into how simple informational transitions between interacting members of a consortium could lead to self-sustaining structured populations comprising proto-autopoietic networks, and could therefore initiate a bridge in the transition from inanimate to living matter via a collective process of protocell self-production operating under non-evolutive/self-replication conditions. Moreover, this in turn might provide a resilient platform for the onset of evolutionary processes responsible, for example, for the emergence of protolife entities from prebiotic inanimate systems. While closed systems based on single causal state automata are incapable of simulating evolution in the Darwinian sense due to the absence of novelty in the automata types produced over successive generations, the functional composition of two-state automata is known to generate communication channels exhibiting entirely novel features [16], suggesting that the environmental dependence of such communities would provide a rich landscape for modelling more complex aspects of autopoiesis. Simulations based on these multi-state systems are the focus of future work.

## Data accessibility

The information niche model has been implemented in Matlab and the code and associated files are available online at https://github.com/rjcarte/Information-Niches.
